# Therapy and Prevention of Noise Fears in Dogs—A Review of the Current Evidence for Practitioners

**DOI:** 10.3390/ani13233664

**Published:** 2023-11-27

**Authors:** Stefanie Riemer

**Affiliations:** Comparative Cognition, Messerli Research Institute, Vetmeduni Vienna, 1210 Vienna, Austria; riemer.stefanie@gmail.com

**Keywords:** anxiolytic medication, behaviour modification, behavioural therapy, counterconditioning, dog *Canis familiaris*, desensitisation, fear prevention, noise phobia, noise sensitivity, relaxation training

## Abstract

**Simple Summary:**

Fear of loud noises is the most common behavioural problem in pet dogs. This manuscript provides an overview of treatment options for noise fears in dogs and describes the current scientific evidence for their effectiveness. Therapy for noise-sensitive dogs involves a combination of management and behavioural training, as well as the potential use of anti-anxiety medication to safeguard dogs’ welfare and prevent worsening of the fear. Providing rewards (food/play) to create positive associations with noises (counterconditioning) is one of the most effective training methods and may change dogs’ emotional responses to noise. Additionally, relaxation training and training with audio recordings can improve noise fears in dogs. While a variety of products to alleviate fear in dogs are on the market, to date, evidence indicates that most “alternative” remedies, such as nutraceuticals, herbal remedies, pheromones, homeopathy, Bach flowers, and essential oils, are not powerful enough to help dogs overcome serious fears. In contrast, the effectiveness of several types of anti-anxiety medications has been demonstrated. Noise fears in puppies and adults can be prevented by creating positive associations with sudden noises.

**Abstract:**

Noise fears represent the most common behavioural problem in dogs. This manuscript provides an overview of diverse approaches for alleviating fear of noises in dogs and the supporting evidence. In the treatment of noise fears, both short-term solutions to prevent trauma or the deterioration of fear during unavoidable noise events and longer-term training need to be considered. Environmental management, the provision of incentives (food/play) during noise exposure, and, when indicated, anxiolytic medication, can safeguard dogs’ welfare during noise events. Most “alternative” products (such as nutraceuticals, herbal remedies, pheromones, homeopathy, Bach flowers, and essential oils) are unlikely to be sufficient as monotherapy for noise fears, whereas there is good evidence for the efficacy of several anxiolytic medications. In the longer term, counterconditioning to real-life noises, relaxation training, and desensitisation/counterconditioning using noise recordings have been shown to improve fear of noises in dogs. Preventative training appears to be highly effective in preventing the development of noise fears in puppies and adult dogs.

## 1. Introduction

Noise fears represent the most prevalent behavioural problem in dogs—studies indicate that between a quarter and half of the pet dog population are affected [1,2,3,4,5]. Fireworks are the most common trigger, followed by thunder and gunshots [4]. Loud noises can have a traumatising effect on dogs, and in some cases, recovery after a single event takes weeks or months [3]. In addition to New Year’s Eve fireworks, fireworks may also occur in the context of other celebrations and can potentially harm animals year-round. Gunshots or bird scare cannons, used to deter birds from crops, are common locally.

Thunderstorms may occur unpredictably and frequently during some seasons, and modelling suggests that as a result of anthropogenic climate change, the incidence of severe thunderstorms will increase [6,7]. Thus, loud noises constitute a significant welfare concern for the pet dog population.

Noise fears often manifest early: a majority of affected dogs show signs already in the first or second year of life [3,8]. Although noise sensitivities often become more severe with age, a new onset of noise fears is relatively uncommon in dogs over six years of age [3]. However, if a dog suddenly develops a fear of noises at an older age, it should be considered that this could also be indicative of a pain issue [9]. In addition to pain, in general, any health condition, such as endocrine diseases, cardiopulmonary disease, neurological problems, and cognitive dysfunction, can potentially contribute to or exacerbate behavioural problems including noise fears [10,11]. A thorough clinical examination and, where indicated, optimised pain management can therefore be important components in the therapy of noise fears [9].

The common early onset of noise sensitivity indicates a genetic contribution. This was also confirmed by differences between breeds or breed groups [1,3,4,5,12] and genetic studies, which consistently indicated within-breed heritability estimates above 0.20 [13,14,15,16] (i.e., high heritability, c.f. [17]).

Nonetheless, two studies found that the “breed group” that scored highest on noise fear were mixed breeds [1,3]. This may hint at the importance of environmental factors. In [3], mixed-breed dogs were adopted from shelters or from the street more often than purebred dogs, which were acquired more often from breeders [3]. It can be speculated that the mixed-breed dogs had less socialisation experiences than the purebred dogs since the purebred dogs were probably more likely than mixed breed dogs to originate from regulated breeding. Official breeders might be more educated about the importance of early socialisation and/or more invested in raising behaviourally sound pups. Indeed, large-scale questionnaire studies demonstrated that after age, the number of experiences during the socialisation period was the factor most strongly associated with extent of fear of fireworks and thunder in pet dogs. More socialisation experiences corresponded to lower noise fears [4,18].

### Treatment of Noise Fears—Considering the Quality of the Evidence

The aim of this review is to summarise recommendations and the current evidence for interventions in the therapy and prevention of noise fears in dogs in view of scientific quality criteria.

The gold standard in clinical research is represented by randomised, double-blind, placebo-controlled studies [19]. A *placebo effect* is defined as “any improvement or change in subjective discomfort or illness resulting from an intervention possessing no physical effect” [20]. A *caregiver placebo* effect in companion animals has been defined as “a sham medical intervention that causes pet caregivers (owners or veterinarians) to believe the treatment they provided to the pet improved the pet’s condition” [21].

In placebo-controlled veterinary studies, 30–50% of owners whose animals received a placebo commonly report improvement [22,23]. In relation to fear responses to fireworks during New Year’s Eve, 37% of owners whose dogs received a placebo reported a good or excellent treatment effect [24].

For this reason, open-label studies must always be interpreted in the context of a likely caregiver placebo effect. Other potential issues include high dropout rates (as owners who perceive the treatments as ineffective are more likely to discontinue the study, c.f. [25]) and drawing conclusions about the efficacy of a product based on one or a few significant effects out of a large number of comparisons without correcting for multiple testing. When using a p-level of 0.05, by definition, we would expect one false positive out of 20 tested variables (c.f. [26]). Furthermore, small sample sizes are less likely to generate robust results, and publication biases may occur because non-significant results are often not published [26].

## 2. First Priority: Dog Welfare and Safety during Noise Events–Management

Therapy for noise-sensitive dogs involves a combination of management (i.e., measures to minimise adverse experiences for the animal), behaviour modification, and, where indicated, anxiolytic medication to safeguard dogs’ welfare and prevent worsening of the fear.

### 2.1. Management Recommendations during Noise Events

Environmental management is considered an important (but not sufficient) component of the treatment of noise fears, with the aim of giving the dog a sense of security and reducing the intensity of fear-eliciting stimuli as much as possible [27]. It is recommended to provide a safe haven for the dog, such as in a comfortable dog crate, that may be covered to block out visual stimuli. This safe space should ideally be associated with relaxation and pleasant experiences before the noise event [27,28]. Note that for animal welfare reasons, keeping dogs in a closed crate is acceptable only for short times after gradual habituation [29], and for the purpose of functioning as a safe space, entering and leaving the crate must always be possible voluntarily.

It can be helpful to stay in a windowless room, or to block out visual stimuli by closing curtains and blinds [30]. When attempting to mask outside sounds, such as with music or white noise, it needs to be considered that the similarity of the masking sound to the noise to be masked is critical for the masking effect, according to research in humans [31]. Thus, a loud fan and repetitive drum beats may be suitable for use with thunder or fireworks [28,30].

Furthermore, while dogs that are very fearful will usually be unable to play or eat during noise events, it is helpful to distract the dog with food, games, or little training tasks whenever this is possible [10,27,30,32]. Ideally, the contingency “loud noise → reward” should be followed. This can enable the dog to form a positive association with the predictor (i.e., the noise) of the reward (c.f. [33,34]).

Contrary to earlier advice, ignoring fearful dogs seeking contact is not advisable. While the data are insufficient in relation to noise fears, there is evidence from other stressful situations that stroking or speaking by a caretaker reduces behavioural and physiological stress indicators in dogs (e.g., [35,36,37]). Of course, such interactions should only be carried out if the dog seeks contact on its own accord—unsolicited touching or even restraint could increase the stress level further.

### 2.2. Feeding and Playing during Noise Events Can Create Positive Associations

Only one study to date [25] assessed the owner-reported effectiveness of different management techniques during real-life noise events. A sample of over 1200 dog owners reported which management they employed during firework exposure to help their dogs. A Principal Components Analysis yielded four components labelled “Environmental modification” (e.g., providing a hiding place, keeping windows and blinds closed, and playing music), “Feed/Play” (providing the dog with chews, play, and food during fireworks as well as contingent on loud bangs), “Alternative” (the use of calming nutraceuticals, pheromones, herbal products, homeopathic products, Bach flowers, and essential oils) and “Interaction” (allowing body contact; petting and talking to the dog when loud bangs occurred)” [25].

When relating each of these four components to a score indicating the degree of improvement or deterioration in the dog’s fear of fireworks, there was no significant relationship between fear progression and the components “Alternative” and “Interaction”. The component “Environmental modification” was associated with a significant deterioration in fear. This is unlikely to indicate that environmental modification is ineffective or even detrimental but could be explained by owners being more likely to perform environmental management if they perceived that their dogs’ fear was severe and had become worse [25].

However, the component “Feed/Play” was associated with a significant improvement in fear scores [25]. This indicates that as long as dogs are still able to eat/play, the use of these incentives can improve their emotional responses to the loud noise [25]. The observed positive effect of feeding/playing on fear progression cannot be attributed to a priori differences between dogs that were still able to eat during noise and those that were too fearful: a re-analysis of the data excluding dogs that refused to eat yielded the same result (Supplementary Material). Note that refusing food is an indicator of high stress [38]; if dogs refuse even high-value food, support with anxiolytic medication may be indicated.

It has been a long-standing belief that attention or food rewards can inadvertently reinforce fear. However, fear is an emotion, not an operant behaviour. Rewards by definition do not make an emotion more negative—on the contrary, the positive experiences can elicit (more) positive emotions, which may subsequently cause a change in behaviour [39]. Thus, offering high-value rewards during noise events to fearful animals is recommendable, even if the animal already shows signs of fear.

## 3. Therapeutic Options for Noise Fears

### 3.1. Calming Products

While “natural” remedies are popular with many dog guardians and are often preferred to prescription medication [40], for most products on the market, there is a lack of high-quality evidence (reviewed in [25]).

Based on an extensive questionnaire survey, Riemer [25] presented a relative comparison of the perceived effectiveness of a large number of therapeutic options for noise fears in dogs (from training approaches to calming products to prescription medication).

In this study, 27% of owners who supported their dogs with nutraceuticals reported that this was effective in alleviating their dogs’ noise fear. This was the lowest success rate of all interventions included. While no conclusions can be drawn regarding individual products from this study, it appears that the effect of the majority of nutraceuticals in use does not exceed that of a (caregiver) placebo effect [25]. Nutraceuticals frequently recommended by veterinary behaviourists include Zylkene^®^ (alpha-casozepine) [41] and L-theanine [30].

The reported effectiveness of herbal products (35.1%), homeopathic remedies (31.2%), Bach flowers (33.5%), and essential oils (31.1%) in [25] was also in the range consistent with a placebo effect. Although individual products were not evaluated, the data (with at least 183 respondents per category) suggest that most nutraceuticals, herbal products, and alternative remedies will likely provide insufficient relief when used as monotherapy for noise fears [25].

#### Individual Studies on Calming Products

*Dog-Appeasing Pheromones (Adaptil^®^)*:

For Dog-Appeasing Pheromones (DAPs)—synthetic pheromones that mimic pheromones produced by nursing bitches (Adaptil^®^ diffuser, spray, or collar)—study findings have been inconsistent. In a blinded, placebo-controlled study exposing laboratory beagles to recordings of thunder, the use of Adaptil^®^ collars was associated with lower active (such as startling, bolting, pacing, and barking) and global fear scores but with a higher incidence of hiding and no significant group differences in passive scores (e.g., freezing, low body postures, panting, trembling) [42]. Other small studies on DAPs reported high success rates but are difficult to interpret as they were open-label and included several interventions at the same time, such as behavioural advice or desensitisation/counterconditioning with CDs alongside the pheromone treatment [43,44,45]. In the larger-scale questionnaire study in [25], out of 316 owners who had used pheromone products to help their dog, only 28.8% considered this treatment to be effective—which is not more than would be expected from a placebo effect [25].

*Fish hydrolysate supplement*:

A placebo-controlled study indicated that the use of a fish hydrolysate supplement was associated with lower cortisol responses and a reduced hyperactivity response to noise recordings in beagles [42].

*Magnolia and Phellodendron extract*:

Another placebo-controlled study on a chewable oral botanical product found no significant treatment or order effect, but a post hoc analysis indicated that a higher number of dogs receiving the product reduced inactivity during a noise recording than in the placebo condition, which was interpreted as demonstrating a benefit of the tablet [46].

Since both increases and decreases in activity have been interpreted as demonstrating an anxiolytic effect [42,46], future studies should measure behavioural expressions that may be more clearly indicative of fear responses (see, e.g., [47,48,49]).

*Homeopathic remedy*:

In a placebo-controlled study, no difference in improvement in firework fears was found between a homeopathic remedy and a placebo [50].

*Cannabidiol* vs. *Trazodone*:

A study comparing the effects of cannabidiol and trazodone (a serotonin antagonist and reuptake inhibitor) on dogs exposed to firework recordings found no evidence for anxiolytic properties of cannabidiol, unlike for trazodone [51].

*L-theanine*:

In an open-label study on the use of an L-theanine supplement for fear of thunderstorms in dogs, the authors of [30] reported a significant reduction in owner-reported global anxiety scores and time to return to baseline after a storm. However, almost one-third of the initial study population (8 of 26 dogs) dropped out prematurely [30], creating a potential bias in the remaining subjects that were available for analysis.

### 3.2. Pressure Vests

Pressure vests are tight-fitting garments assumed to have calming effects from exerting deep pressure [52]. There are some indicators that pressure vests may have the potential to lower physiological or behavioural signs of anxiety in dogs [53,54,55]. It must be considered, however, that the handling associated with putting on the vest, wearing the tight vest itself, or the Velcro noise may potentially be stressful for some dogs.

When opting to use a pressure vest to reduce a dog’s anxiety, it is therefore important to habituate the dog to wearing it before the event and to use it repeatedly outside of fearful situations [52]. A systematic review on the available studies suggests that a small beneficial effect is possible [52]. In [25], the reported success rate for pressure vests was 44%, which was higher than for all other non-pharmaceutical products. Thus, some individual dogs might benefit from wearing a pressure vest [25].

### 3.3. Psychopharmacology

Pharmacological treatment may be indicated to relieve the immediate distress of dogs during noise exposure and potentially to increase the success of behavioural modification in which the dog learns a new association with the former trigger [56]. Indeed, in the comparative study by Riemer [25], the low success rates for non-pharmaceutical products are in contrast to the higher effectiveness reported for prescription medication. Even though we can assume that the dogs that were prescribed medication were on average more fearful than those that were not, the success rate was 68.9% for anxiolytic medication and thus (more than) double than that of the aforementioned products evaluated in the study, except for pressure vests [25].

#### 3.3.1. Short-Term Medication Options

*Sileo^®^*:

The effectiveness of Sileo^®^ oral dexmedetomidine gel has been demonstrated in a placebo-controlled, double-blinded clinical field study [24]. Over seventy-one percent of owners from the treatment group reported a good to excellent effect of the drug during New Year fireworks—significantly more than in the placebo groups [24]. In [25], the reported success rate for Sileo^®^ was similar to in [24] at 74%; in [57], it was 61%.

There is even evidence indicating that the repeated administration of Sileo^®^ during different types of noise events enabled dogs to learn to cope better with noises so that the use of the medication could be reduced [58].

*Pexion^®^*:

A placebo-controlled, randomized, double-blinded clinical trial indicated that treatment with Pexion^®^ (imepitoin), starting two days before New Year’s Eve, was associated with significantly reduced signs or fear and anxiety during New Year’s Eve fireworks compared to a placebo. Accordingly, approximately two-thirds of owners whose dog received Pexion^®^ reported a good to excellent treatment effect—significantly more than in the placebo group [59]. The regular twice-daily administration of Pexion^®^ was also found to decrease anxiety during thunderstorms in a double-blinded placebo-controlled study [60].

*Gabapentin*:

In a double-blinded cross-over trial, gabapentin was associated with significantly lower fear scores during thunderstorms in dogs, although three of eighteen subjects had higher fear scores on gabapentin than when given a placebo [61].

*Trazodone* vs. *Sileo*:

A smaller study compared the effects of Trazodone with those of Sileo^®^ on New Year’s Eve (no placebo group) and found both medications to be effective. However, the reduction in fear scores and owner satisfaction were significantly greater for Trazodone (87.5% of owners satisfied) than for Sileo^®^ (61.1% of owners satisfied) [57].

*Alprazolam*:

The reported effectiveness of alprazolam for firework fears was 90.6% in [25].

*Clonidine*:

In a small open-label study on clonidine, which included dogs affected by separation distress as well as noise or storm phobia, positive outcomes were reported by 70% of owners [62]. However, according to practitioner reports, success for noise fears with clonidine is variable [63] (see also a reported lack of effect on firework fears in the two dogs treated with clonidine in [25]).

*Acepromazine*:

Acepromazine is contraindicated for noise fears [10,56,64]. As a tranquiliser and dissociative agent, it appears to have no anxiolytic properties [64,65,66] and is even suggested to heighten sound sensitivity [10,56,64].

In many cases, a combination of psychoactive drugs is more effective than a single substance. However, careful consideration of the interactive effects on drug metabolism must be given [67]. Since individual animals may react very differently to psychoactive drugs, the treatment needs to be tailored to the individual and should be trialled before the event.

#### 3.3.2. Longer-Term Medication Options

While the right medication for a given animal can be effective, it requires the owner to administer it on time (i.e., before the noise starts or panic sets in [27]), which can be challenging for events that cannot be planned. If an animal responds well to Pexion^®^, this is an option that could also be given over a longer time period, e.g., during the thunderstorm season (c.f. [60,68]). Other longer-term anxiolytic medications can be indicated for some cases, but they have not been tested specifically for noise fears in dogs. Examples include MAOIs (monoamine oxidase inhibitors) and SSRIs (selective serotonin reuptake inhibitors) [27,41,64]. Potentially, these may need to be combined with a short-acting anxiolytic, such as dexmedetomidine or a benzodiazepine, on an as-needed basis [63]. For instance, one study reported improvement in storm phobia in 30 of 32 dogs receiving a combination of daily clomipramine and alprazolam before noise events, as well as desensitisation/counter-conditioning with audio recordings of thunderstorms [69].

As McPeake et al. [63] point out, the optimal medication for a given patient should be selected based on his/her behavioural history (in particular, other possible fear issues) and physical health. Medication should not constitute the sole treatment component, but behavioural management advice should be given, and whenever possible, the underlying fear should be addressed via a behaviour modification plan [63].

Consultation with a specialist in veterinary behavioural medicine can be valuable for making decisions about psychopharmacological treatments, especially where combinations of medications are concerned, when the animal’s behaviour is unusual, or when previous treatment attempts have not led to sufficient improvement.

### 3.4. Behaviour Modification

In the longer term, behavioural training (possibly in conjunction with pharmacological treatment to support learning, depending on the extent of the dog’s phobia) is important for modifying a dog’s emotional response to a fear-evoking situation [56].

#### 3.4.1. Desensitisation and Counterconditioning Using Noise Recordings

*Desensitisation* refers to the gradual and progressive presentation of the fear-eliciting stimulus, starting at a level that does not elicit anxiety, to allow the dog to learn that the stimulus is harmless. Over the course of numerous repetitions, stimulus intensity can be gradually increased, and the initial fear response can be extinguished [39,70].

*(Classical) counterconditioning* refers to pairing the fear-eliciting stimulus with desirable consequences (such as food or play) in order to change the association with the stimulus [71]. Counterconditioning is most effective if the trigger is reliably followed by a reward each time and when combined with desensitisation [39].

The use of recordings of fireworks or thunderstorms for desensitisation and counterconditioning has been considered the gold standard in the therapy of noise fears [43,45,72]. An example protocol for desensitisation/counterconditioning is given in Rogerson [38]. The author also highlights the importance of the quality of the rewards used and presenting the stimuli in a carefully graded manner. He furthermore reminds the reader to take into account all sensory channels (such as smells, sights, or atmospheric pressure in addition to the noise) [38]. When the dog has improved, intermittent training is recommended to avoid relapses [43,45].

Some studies indicated high success rates from training with noise CDs [44,45] (however, see [44,69] for a lack of improvement in objective measures, contrary to improvements reported by the owners). In the study with the largest sample size so far, the reported success rate for noise recordings was 54.5% and thus lower than for other training approaches, counterconditioning (to real-life noise) and relaxation training [25] (see below). On one hand, the time and expertise needed for successful desensitisation may represent an obstacle [73]; on the other hand, recordings do not display the full frequencies of the actual noise, and lights and other sensory stimuli that are associated with fireworks or thunderstorms are missing [74]. Even under optimal acoustic conditions, some dogs that react fearfully to real-life noise events show no reaction to recordings [74].

While recordings are thus a great way to expose dogs to noises in a controlled manner, at an intensity that can be adjusted such as not to elicit any fear response, not every fearful dog appears to benefit from training with recordings.

#### 3.4.2. Ad Hoc Counterconditioning

Fear extinction is most effective when the eliciting stimulus can be presented carefully and gradually, as described above [39]. However, it is often impossible to control loud noises in real life. In such cases, “ad hoc counterconditioning” (e.g., giving a treat, playing with the dog, or celebrating a little “party”) whenever a loud noise occurs can nonetheless contribute to an improvement in firework fears in dogs [25]. Thus, not only was feeding and playing with the dog during firework events the only one of four management strategies that was associated with a significant improvement in noise fears in [25]: When asked directly about the perceived effectiveness of different training approaches, over 70% of respondents indicated that counterconditioning was successful in alleviating their dogs’ noise fears, making it the most successful intervention in the study [25].

#### 3.4.3. Relaxation Training

Relaxation training is a lesser known, yet effective method in behavioural modification [25,75]. Briefly, the animals learn to relax on cue, for which various training approaches exist. One method is based on classical conditioning. Relaxation is induced, such as by stroking or massaging the dog, or it can be captured when the dog is relaxed on its own. If relaxation can be reliably induced or predicted, it can be paired with a signal. For instance, a particular word might be repeated, or a particular blanket could be used for massage sessions. It is also possible to use a scent (e.g., lavender oil), music, or audiobook as a signal. After successful conditioning, the signal can be used to elicit relaxation/reduce arousal (c.f. [41]).

An alternative approach is the operant reinforcement of relaxed behaviour. Overall [53] created a “protocol for training relaxation”, which includes a stay exercise in the face of increasing distractions. Additionally, she recommends specifically rewarding behaviours and facial or bodily expressions consistent with relaxation [53]. Ballantyne [76] suggests either rewarding spontaneous relaxed behaviour by the dog or using a step-by-step training plan, starting with rewarding the dog for approaching, then lying on a mat, and eventually the targeted reinforcement of relaxed behaviors such as resting the head, rocking the hip to one side, extending the hind legs from the body, and lying on the side [76].

In [25], 69% of owners who used relaxation training reported that it helped their dog—this success rate is comparable to those of counterconditioning and anxiolytic medication and higher than the success rate for training with noise recordings in this study.

## 4. The Prevention of Noise Fears Is Possible!

Generally, rich experiences during the socialisation period are associated with a lower incidence of noise fears [4,18]. Short-term positive effects on startle responses and recovery after a loud noise were demonstrated by a training programme for young puppies (3–6 weeks) involving targeted exposure to sudden stimuli of increasing volume, subject to the puppies’ relaxed responses. However, no long-term effects were found, possibly because the training programme stopped early in the socialisation period [77].

An important role of the guardians’ training efforts was demonstrated in [3]: the preventative training of both puppies and adult dogs appears to prevent the development of noise fears, probably by enabling the animals to create positive associations with noises [3]. This appears to be particularly effective during the puppy stage, but also in adult dogs, training before the dog showed any signs of fear was associated with significantly lower fear scores. On a Likert scale ranging from one (welfare not compromised by fireworks) to five (welfare strongly compromised by fireworks), the median score for dogs trained as puppies was one and for those trained as adults, it was two, whereas no preventive training was associated with a median score of four [3].

Ad hoc counterconditioning whenever a loud noise occurs is easy to implement in everyday life and is effective in preventing and improving noise fears in dogs [25]. Since dogs respond to our emotions and seem to adapt their perception of a situation based on humans’ emotional communication [78], it is also recommendable to show signs of positive emotions whenever potentially startling stimuli happen.

## 5. Conclusions

In the best case, noise fears in dogs can be prevented via preparatory training. In dogs already affected by noise fears, management, providing incentives (food/play) during noise events, and the potential use of anxiolytic medication can prevent trauma during unavoidable noise events and safeguard the dogs’ welfare. In the long term, training should enable dogs to learn a new positive association with the fear-eliciting stimulus. In addition to the commonly recommended use of noise recordings, counterconditioning (even ad hoc; any loud noise is followed by a reward) and relaxation training were demonstrated to be particularly effective in alleviating dogs’ fears.

## Data Availability

Not applicable.

## Supplementary Materials

The following supporting information can be downloaded at https://www.mdpi.com/article/10.3390/ani13233664/s1, Re-analysis of the relationship between fear progression and feeding/playing during fireworks from Riemer (2020), excluding dogs that were reported to refuse food during firework events.
