# Supplementary material for: Therapy and Prevention of Noise Fears in Dogs—A Review of the Current Evidence for Practitioners
Source: Animals (Basel). 2023 Nov 27;13(23):3664. doi: 10.3390/ani13233664 (PMC10705068; doi:10.3390/ani13233664)
Supplement: Supplementary file 1 [file animals-13-03664-s001.zip › animals-2701034-supplementary.pdf]

animals

MDPI

Therapy and Prevention of Noise Fears in Dogs—A Review of the Current Evidence for Practitioners

Stefanie Riemer

Comparative Cognition, Messerli Research Institute, Vetmeduni Vienna, 1210 Vienna, Austria; riemer.stefanie@gmail.com

Citation: Riemer, S. Therapy and the Prevention of Noise Fears in Dogs—A Review of the Current Evidence for Practitioners. *Animals* **2023**, *13*, 3664. https://doi.org/10.3390/ani13233664

Academic Editor: Mandy Paterson

Received: 20 October 2023
Revised: 14 November 2023
Accepted: 23 November 2023
Published: 27 November 2023

Copyright: © 2023 by the author. Licensee MDPI, Basel, Switzerland. This article is an open access article distributed under the terms and conditions of the Creative Commons Attribution (CC BY) license (https://creativecommons.org/licenses/by/4.0/).

Supplementary Material:

1. Background

A reviewer of the manuscript "Therapy and prevention of noise fears in dogs – a review of the current evidence for practitioners" suggested that an alternative explanation for the finding from [1] that owners' feeding and playing with the dog during fireworks was associated with more favourable fear progression could be that dogs that were willing to play or eat during fireworks were less fearful to begin with.

I was able to analyse this question since the questionnaire used in [1] also included questions on dogs' behavioural signs during fireworks (unpublished data).

2. Methods

The Feed/Play component was one of four principal components resulting from a nonlinear Principal Components Analysis over 21 questions on owners' management strategies during fireworks. It had loadings for five variables: providing food, giving chews, and playing during fireworks, as well as reacting to bangs by feeding and playing with the dog [1]. For full details, see [1].

The fear progression score was rated on a five-point Likert scale from 1 to 5 (1—"The fear has improved greatly;" 2—"The fear tends to have improved;" 3—"The fear has remained the same;" 4—"The fear tends to have become worse"; 5—"The fear has become much worse")[1].

The question whether the dog refused food during fireworks was also answered on a Likert scale ranging from 1 to 5 (1—"disagree strongly;" 2—"tend to disagree;" 3—"partly/partly;" 4—"tend to agree"; 5—"agree strongly").

I re-calculated the Spearman correlation between the Feed/Play component and the fear progression score from [1], but I included only dogs with scores 1 and 2 on the "Refuse food" variable in the analysis, i.e., dogs that were able to eat during fireworks.

3. Results

The association between the Feed/Play component and an improvement in firework fears was significant also in this subsample (r_S=-0.19, p=0.0044).

4. Discussion

The results confirmed the findings from the original study that fear progression was significantly negatively correlated with the Feed/ Play component, i.e., more feeding/ playing with the dog during fireworks was associated with fear improvement.

While the most fearful dogs will likely be unable to eat or play during fireworks, the positive effect of feeding/playing in [1] cannot be attributed to a biased sample of dogs that were still able to eat as the relationship between improvement and Feed/Play was equally strong (even slightly stronger) in the subset of dogs that were willing to eat during fireworks (rS=-0.19 as opposed to -0.16 in [1]).

Reference

1. Riemer, S. Effectiveness of Treatments for Firework Fears in Dogs. *Journal of Veterinary Behavior - Clinical Applications and Research* **2020**, *37*, 61-70.
